# How to build an inducible cartilage-specific transgenic mouse

**DOI:** 10.1186/ar4573

**Published:** 2014-06-02

**Authors:** Esmeralda N Blaney Davidson, Fons AJ van de Loo, Wim B van den Berg, Peter M van der Kraan

**Affiliations:** 1 Department of Experimental Rheumatology, Radboud University Medical Center, 272 Geert Grooteplein 26-28, 6500 HB, Nijmegen, The Netherlands

## Abstract

Transgenic mice are used to study the roles of specific proteins in an intact living system. Use of transgenic mice to study processes in cartilage, however, poses some challenges. First of all, many factors involved in cartilage homeostasis and disease are also crucial factors in embryogenesis. Therefore, meddling with these factors often leads to death before birth, and mice who do survive cannot be considered normal. The build-up of cartilage in these mice is altered, making it nearly impossible to truly interpret the role of a protein in adult cartilage function. An elegant way to overcome these limitations is to make transgenic mice time- and tissue-specific, thereby omitting side-effects in tissues other than cartilage and during embryology. This review discusses the potential building blocks for making an inducible cartilage-specific transgenic mouse. We review which promoters can be used to gain chondrocyte-specificity - all chondrocytes or a specific subset thereof - as well as different systems that can be used to enable inducibility of a transgene.

## Building an inducible cartilage-specific transgenic mouse

When researching cartilage, one will soon enough encounter the limitations involved when studying cartilage and chondrocyte behavior *in vivo*. In this non-innervated tissue, which is extremely hard for many proteins to penetrate due to size and/or charge, it is very difficult to target the chondrocytes. In addition, cartilage is affected by many secondary effects from surrounding tissue. Meddling with factors involved in chondrogenesis will alter cartilage composition, so when studying cartilage diseases one requires build up to be normal, thereby omitting the option of a 'classical' transgenic mouse. A way to overcome this problem is to generate an inducible cartilage-specific transgenic mouse. To drive a transgene of interest in the desired tissue, one has to decide which promoter to choose as this will define the expression pattern. Thereafter, the system to induce expression of the transgene has to be defined. This review discusses the different promoters and inducible systems most commonly used.

### Chondrocyte-specific promoters

Cartilage is a highly specialized tissue consisting of an extracellular matrix mainly composed of aggrecan and collagens. These molecules are specifically expressed by the chondrocytes and the driving forces (promoters) leading to their expression can therefore be used to facilitate chondrocyte-specific expression of transgenes. A good promoter should enable expression of a transgene in the desired cell, in this case chondrocytes. There are many types of chondrocytes, however, so the choice of promoter can determine whether expression occurs in chondrocytes in general or in a specific subset thereof.

A good promoter is generally perceived as one that results in the highest possible expression of a transgene to give a solid phenotype. Therefore, the most commonly used promoters are the result of studies identifying crucial elements for high transgene expression.

The collagen type II promoter and the collagen type XI promoter are widely used. The aggrecan promotor is less commonly used, most likely due to its complex regulation. When chondrocytes undergo hypertrophy, collagen type X is a typical marker and as such can be used to specifically target hypertrophic chondrocytes. Matrillin, which is secreted by chondrocytes and involved in making extracellular matrix networks, is a less evident marker. Although *Gdf5* is not specific for cartilage, it is important in new joint formation and its promoter might therefore still be an interesting choice depending on experimental requirements. Collagen type VI is common in connective tissue, but highly present in the pericellular chondrocyte matrix. The *Prx1* (paired related homeobox 1) promoter is used to study mesenchymal cells, the precursors of chondrocytes, and is therefore included in this review as well. All of these potential promoters are discussed below.

#### ***Col2a1***

Collagen type II is an early marker for chondrogenesis and is expressed from embryonic day (E)9.5 in mouse in cranial mesenchyme destined to develop into the chondrocranium and in sclerotome of the somites [1]. The collagen type II gene (*Col2a1*) is expressed in all chondrogenic tissues of the axial and appendicular skeleton until onset of endochondral ossification [1]. However, it is also expressed transiently at low levels in certain non-chondrogenic tissues, including notochord, eye, heart, epidermis and discrete areas of the brain [1]. The *Col2a1* promoters target chondrocytes and perichondrium as well as the mesenchyme near presumptive joints. It is not until E12.5 to E13.5 that its expression becomes predominantly restricted to chondrocytes.

##### 

**Regulatory elements in the *Col2a1 *promoter and their effect on expression** In 1995 Zhou and colleagues [2] investigated chondrocyte specificity of the *Col2a1* promoter in murine embryos. They made promoter and intron 1 constructs of various lengths linked to a beta-galactosidase reporter. This showed that a 3,000 bp promoter and 3,020 bp intron 1 construct induced high levels of chondrocyte-specific expression, coinciding with temporal expression of the endogenous gene. Deletion studies revealed that a 182 bp fragment within intron 1 was as specific as the entire 3,020 bp intron 1 fragment. Specifically, this 182 bp fragment was crucial for targeting expression to chondrocytes as replacing the *Col2a1* promoter fragment with that of beta-globulin promoter resulted in similar targeted expression. The only difference was that the beta-globulin promoter additionally showed expression in the brain. Expression in the midbrain of murine embryos has been suggested to rely on specific positive and negative regulatory elements upstream and downstream of the *Col2a1* promoter [3]. Mukhodpadhyay and colleagues [4] confirmed the importance of intron 1 of *Col2a in vitro* in a rat chondrosarcoma (RSC) cell line, where serial deletions of the promoter region led to decreased activity; these deletions could all still be activated when combined with a 231 bp intron 1 fragment including the 156 bp enhancer. Similar to Zhou and colleagues, they showed that combining the intron 1 fragment with a different promoter still drove chondrocyte-specific expression. For optimal promoter activation, interactions between enhancer-bound proteins and proteins bound to the promoter segment between positions -89 and +6 was required. However, no *Col2a1* promoter-specific elements were required for activity as the promoter could be substituted for another promoter without affecting specificity. Using four copies of the 48 bp intron 1 fragment linked to a 309 bp *Col2a1* promoter, Lefebvre and colleagues [5] observed a perfect cartilage-specific expression pattern in mice. They suggest that elements inhibiting expression in brain and possibly skin might be located in the promoter between positions -309 and -89 or in the 48 bp element outside the 18 bp enhancer. Of great interest is that the enhancer in intron 1 that drove chondrocyte specificity in the RSC cell line contained a binding site for Sox9 [6].

##### 

**Use of the *Col2a1 *promoter *in vivo*** Currently, the commercially available *Col2a1*-cre mouse described by Ovchinnikov and colleagues [7] is the most commonly used. The cre-loxP system will be discussed later in this review. This *Col2a1*-cre construct is composed of 3 kb of the *Col2a1* promoter region, the first exon with a mutated initiation codon, a 3.02 kb fragment of intron 1 ligated to a splice acceptor sequence, followed by an internal ribosome-entry site (IRES), cre recombinase and SV40 large T antigen polyadenylation signal. Breeding these mice with the ROSA26 cre reporter strain showed activity in notochord and cranial mesenchyme between 8.75 and 9.0 days post-coitum (dpc), activity in somites at 9.5 dpc, and intense beta-gal staining in vertebral anlagen undergoing chondrogenic differentiation by 11.5 to 12 dpc. Later in development, specifically cartilaginous tissues stained for beta-gal activity. There was no staining in osteoblasts or perichondrial fibroblasts. Sakai and colleagues [8] made a highly similar *Col2a1*-cre fusion construct that was expressed in notochord, developing brain, sclerotome and mesenchymal condensations of future cartilage (E9.5 to 11.5). At later stages they showed expression in all cartilaginous tissues. Belteki and colleagues [9] used a *Col2a1*-cre mouse with floxed vascular endothelial growth factor-A and showed high cre recombinase levels in epidermis, eye and heart. Sakai and colleagues did not find expression in the heart and Ovchinnikov and colleagues did not describe expression in nervous system and heart. This shows that each version of a promoter, even if it seems highly similar to others, requires careful investigation of expression patterns as each small alteration can alter tissue specificity.

#### ***Col11a2***

Collagen type XI consists of three subunits and plays a crucial role in regulating formation of collagen fibrils. Whereas the α1(XI) and α3(XI) genes are expressed in many tissues, the α2(XI) gene in mice is more restricted to cartilage [10].

##### 

**Regulatory elements in the *Col11a2 *promoter and their effect on expression** In 1996 Tsumaki and colleagues [11,12] studied collagen α2(XI) (*Col11a2*) expression and characterized which regulatory elements were required for chondrocyte/cartilage-specific expression *in vivo*. They showed that the upstream 742 bp and intron 1 were crucial for expression in primordial cartilage. Shortening the promoter region resulted in less active and more variable expression patterns amongst founder embryos. Deletion of the upstream 290 bp of the 742 bp construct resulted in divergent expression as the primordial cartilage of carpals, tarsals and vertebral bodies no longer expressed LacZ that was cloned downstream of the promoter, whereas notochord and other primordial cartilage were unaffected [11]. Intron 1 was crucial as it contained an enhancer element important for strong chondrocyte-specific expression, which was also investigated in mouse and chick embryo chondrocytes derived from sterna of 15-day-old embryos [2,13]. This was confirmed by Liu and colleagues [14], who used electrophoretic mobility shift assays (EMSAs) with RCS versus Balb/3 T3 and undifferentiated ATDC5 cells to show that this was mediated through SOX9 transcriptional activation. Tsumaki and colleagues [11] identified four different cell-specific elements in the *Col11a2* promoter: an element between -742 and -453 bp promoting high level gene expression in most chondrocytes; an element within the 2.3 kb intron 1 necessary for high level expression in notochord and nucleus pulposus; an element in intron 1 directing high expression in most chondrocytes in cooperation with the -453 bp promoter; and finally an element in which intron 1 cooperated with an element between positions -742 and -453 that specifically directed expression in primordial cartilage of carpals, tarsals and vertebral bodies [11]. Later, others identified that a 1,064 bp promoter fragment resulted in expression not only in chondrocytes, but also in osteoprogenitors in mouse embryos [15]. Tsumaki and colleagues [16] showed that, depending on the deleted region of the *Col11a2* promoter, it was possible to activate expression in neural tissues or even other random tissues, thereby further discriminating between a cartilage-specific element (-501 to -530) and a neural tissue-specific element. In 2005 Fujimuki and colleagues [17] reported that their *Col11a2*-cre mouse did not show expression in non-cartilage tissues, including heart, brain and osteoblasts except for the notochord. They stated that the expression level of *Col2a1* is higher, but that the *Col11a2*-cre mouse showed higher specificity, potentially due to the lower expression levels.

##### 

**Comparison between *Col2a1 *and *Col11a2*** Comparing the crucial elements of *Col2a1* and *Col11a2* using EMSAs in RCS and BALB/3 T3 cell lines showed that enhancer elements of both genes contained several sites with homology to the high mobility group (HMG) protein-binding consensus sequence [18]. The DNA-protein complex formed by the *Col11a2* elements was dependent on the presence of HMG-like sites and had the same mobility as the complex formed with the *Col2a1* enhancer. The proteins within the complex were the same or similar and both included SOX9. Zhou and colleagues [19] used nuclear extracts from a wide variety of cell types of human, rat and murine origin to show, using EMSAs, that SOX9 binds strongly to the 48 bp *Col2a1* intron 1 sequence and bends it at this site, strongly activating the 48 bp enhancer as well as larger *Col2a1* enhancer elements. Deletion of the HMG-like sites abolished activity whereas ectopic SOX9 expression could even activate the enhancers in non-chondrocyte cells [18,20].

#### ***Agc1***

In contrast to the vast amount of knowledge on the regulation of *Col2a1* and *Col11a1* promoters, aggrecan regulation has been far less researched. Sekiya and colleagues [21] showed that SOX9 induced threefold upregulation of an *Agc1* reporter in a cartilage-derived cell line. Doege and colleagues [22] analyzed the cartilage-specific *Agc1* promoter for chondrocyte regulation purposes *in vivo*. They analyzed transcriptional regulatory elements and found a 4.7 kb DNA fragment with cell-specific enhancer activity approximately 12 kb upstream of the transcription start site. It strongly stimulated *Agc1* expression in chondrocytes and weakly suppressed transcription in fibroblasts. This enhancer was crucial for expression in embryonic cartilage. Without it, neither the short nor a long promoter was capable of inducing expression. Han and Lefebvre [23] studied the *Agc1* promoter using the same 4.7 kb region and showed that it had a highly conserved enhancer directing specificity of aggrecan expression in embryonic and adult cartilage. This enhancer was a direct target of the SOX trio, where SOX9 could only bind in the presence of L-SOX5/SOX6. A specific non-exonic region they called A1 showed the same expression pattern as *Agc1*, but was distinctly different from *Col2a1* reporters in that it was not expressed in skeletogenic mesenchymal cells, perichondrium and presumptive joint cells, but was strictly specific for differentiated chondrocytes. Moreover, it remained active through adulthood whereas *Col2a1* was less active upon ageing. This was speculated to be due to a *cis*-acting element in A1 ensuring lifelong activation by the SOX trio. This would make this A1 region an interesting tool for expressing transgenes in differentiated chondrocytes in adulthood, which was exactly what Henry and colleagues did in 2009 [24]. They generated a tamoxifen-inducible *Agc1* dependent cre recombinase-expressing mouse that also had an X-gal reporter (discussed below). This mouse showed *Agc1* expression in growth plate, articular cartilage, fibrocartilage of the menisci, trachea and intervertebral discs, which reproduced the pattern of endogenous aggrecan gene expression.

#### ***Matn1***

Matrilins (MATNs) are non-collagenous proteins forming collagen-dependent and -independent filamentous networks in the extracellular matrix. They are expressed during limb development, suggesting a function in endochondral bone formation [25]. MATN1 and -3 show skeletal expression mainly in cartilaginous tissues during growth. MATN2 is found in proliferating and hypertrophic cartilage, periosteum and bone, but also in many non-skeletal tissues [26]. MATN4, like MATN2, is abundantly present in a variety of tissues [27]. MATN1 binds to aggrecan [28] as well as collagen type II [29]. It has the most restricted expression pattern and, except for a few non-chondrogenic tissues, is secreted only in hyaline cartilage and during developmental stages of chondrocytes [30]. Furthermore, it is continuously expressed in tissue that remains cartilaginous throughout life [25].

##### 

**Regulatory elements in the *Matn1 *promoter and their effect on expression** Karcagi and colleagues [30] analyzed the *Matn1* promoter and showed that both long and short forms governed cartilage-specific activity, but the short promoter showed expression in neural and other tissues as well, depending on the integration site. Both the upstream promoter and intronic sequences were important for driving cartilage-specificity and specific regulatory regions were identified with dispersed cartilage-specific control elements: the promoter upstream region (-2,011 to -338) the promoter (-338 to +67) and the intronic region (+67 to +1,819). Rentsendorj and colleagues [31] showed that transgene expression was highest in columnar proliferating and pre-hypertrophic chondrocytes when the *Matn1* promoter between -338 and +67 was used and very weak in chondrocranium, axial and appendicular skeleton. Similar to the enhancer elements for *Col11a1* and *Col2a1* located in intron 1, the proximal promoter element contained an element interacting with SOX9, which was crucial for tissue specificity. The proximal promoter element of *Matn1* seemed to function at a certain distance from the TATA box, making it clearly different from cartilage-specific enhancer elements of other cartilage protein genes. It was suggested not to function as an enhancer, but rather as a modulator of promoter activity mediating the effect of distal promoter and intronic enhancer elements. Regulation of the *Matn1* gene in mice shared similarities with that of the *Col11a2* gene, with *cis*-elements directing reporter gene expression to specific chondrogenic- and non-chondrogenic tissues [30]. MATN expression is not constant and is subject to change, most likely due to its complex regulation where many positive and negative elements allow independent spatial and temporal regulation in various forms of cartilage [30]. Therefore, identifying and isolating the distinct regions controlling chondrocyte specificity within specific tissues are crucial steps in using the *Matn1* promoter for development of cartilage-specific transgenics. Kargaci and colleagues [30] suggested two strategies for controlling gene expression in chondrocytes, one using the compact enhancer element of *Col2a1* with uniform high chondrocyte activity and the other a combination of several weaker enhancers with distinct functions in subsets of chondrocytes and tissues, such as *Col11a2* and *Matn1*.

#### ***Col10a1***

Whereas *Col2a1*, *Col11a1*, *Agc1* and *Matn1* promoters are used to target young and mature chondrocytes, it is also possible to target chondrocytes specifically near the final stages of differentiation - chondrocyte hypertrophy - by using the collagen type X (*Col10a1*) promoter. This can be used to interfere with endochondral bone formation.

##### 

**Regulatory elements in the *Col10a1 *promoter and their effect on expression** In a comparison of a 4.7 and a 1.6 kb *Col10a1* promoter, only the former led to expression in the endochondral skeleton, whereas the latter showed expression in other organs [32]. Campbell and colleagues [33] used four different transgene constructs for *Col10a1* containing various combinations of different elements: a 4.7 kb or 1.6 kb *Col10a1* promoter fragment in combination with two different chick *Col10a1* transgenes with in-frame deletions. The frame-deleted transgene dominantly interfered with the endogenous collagen X in hypertrophic cartilage, growth plates and ossification centers, showing that the *Col10a1* promoter could be used for tissue-specific overexpression in hypertrophic chondrocytes.

Gebhard and colleagues [34] identified a 12-O-tetradecanoylphorbol 13-acetate-responsive element located in the human *Col10a1* enhancer that was essential for high transcriptional activity and highly conserved also in murine and bovine *Col10a1* genes. This specific enhancer element was necessary and sufficient to drive *Col10a1* reporter gene expression in hypertrophic cartilage in mice *in vivo*. Without the enhancer, the *Col10a1* promoter including intron 1 was not capable of driving tissue-specific expression.

Later, Gebhard and colleagues [35] created reporter mice lines that expressed LacZ in hypertrophic chondrocytes under control of the *Col10a1* promoter to enable analysis of effector gene functions in hypertrophic cartilage. They showed strong expression in chondrocytes in hypertrophic zones and no activity in non-hypertrophic chondrocytes or in non-chondrogenic tissues. The same construct was cloned in combination with cre recombinase to express cre recombinase in hypertrophic chondrocytes as a useful tool to study hypertrophy and endochondral ossification *in vivo*[36,37].

The *Col10a1* promoter contains an activator protein 1 site in the proximal promoter and several RUNX2 (Runt-related transcription factor 2) binding sites, which corresponds to the hypertrophic phenotype *in vivo*[38]. The 150 bp *Col10a1* distal promoter is sufficient to drive hypertrophic chondrocyte-specific expression *in vivo*, which is RUNX2 dependent [39]. Interestingly, even though SOX9 represses RUNX2 expression, depending on the choice of regulatory region, the *Col10a1* promoter might still be SOX9 dependent due to a conserved binding site for SOX9 located at -186/-169 that enhances expression *in vivo*[40].

#### ***Col6a1***

Collagen type VI is common in most connective tissues. Its regulation is rather elaborate. In cartilage, collagen type VI is almost exclusively in the pericellular matrix [41]. In 1996 Braghetta and colleagues [42] investigated the *Col6a1* promoter to see which regions were important for tissue specificity. They showed that different proximal promoter regions were responsible for expression in different tissues, varying, for example, between mesenchymal cells at specific aponeurosis insertion sites and nerves, to subepidermal mesenchyme and muscle. The region -4.0 and -5.4 kb from the transcription start sites was also (but not exclusively) responsible for expression in joints and intervertebral disks. In 1997 Braghetta [43] specified the core of the promoter (-215 bp) and located upstream activator and repressor areas, some of which influenced tissue specificity *in vivo*. Carefully choosing the promoter region would make the *Col6a1* a potential promoter of choice to target expression to joint cartilage, but at present its expression is too elaborate to use it as a cartilage-specific promoter.

#### ***Gdf5***

With regard to targeting chondrocytes, the *Gdf5* promoter is very different from the promoters described above. It is expressed in the developing joint, but is not restricted to meniscal and articular cartilage. Rountree and colleagues [44,45] used the murine *Gdf5* promoter with cre recombinase to target developing joints as GDF5 (growth and differentiation factor 5) expression is specific and limited to sites of new joint formation during embryogenesis. Blocking GDF5 even prevented formation of joints [45]. They showed that *Gdf5*-cre resulted in reporter expression in all structures of mature synovial joints, including ligaments, synovial membrane and articular cartilage [44]. In 2008 Koyama and colleagues [46] showed that *Gdf5*-expressing cells give rise to most if not all joint tissues, including articular cartilage, ligaments and inner synovial lining, but do not contribute to adjacent long bone cartilaginous shaft and growth plate formation. In addition, *Gdf5*-cre also resulted in alterations in intervertebral disc upon transgene expression [47]. The *Gdf5* locus in these experiments was cloned from a bacterial artificial chromosome (BAC) containing the *Gdf5* locus. The necessary promoter and enhancer element in this promoter were not specified.

The use of the *Gdf5* promoter to drive expression circumvents embryonic lethality, but does confer the chondrocyte specificity that most of the other discussed promoters do. However, the *Gdf5* promoter can be very useful when chondrocyte specificity is not required but joint specificity is.

#### ***Prx1***

*Prx1* has been used to study chondrogenesis and/or skeletal limb formation. Its promoter is not at all chondrocyte-specific, but since it is used to study limb formation from mesenchymal cells, the precursors of chondrocytes, it is worth mentioning it here. *Prx1* stands for paired-related homebox gene-1 and was formerly named *MHox1*. It is expressed in the early limb bud skeletal mesenchyme. In transgenic mice a regulatory element in the 2.4 kb genomic flanking region of the *Prx1* gene was sufficient to drive expression in limb bud skeletal mesenchyme and a subset of craniofacial mesenchyme [48]. This region contained a 530 bp core element necessary for expression. Using this *Prx1* limb enhancer with a floxed cre reporter showed first activity at 9.5 dpc in the earliest limb bud and by 10.5 dpc essentially in all skeletal mesenchymal cells in the limb [49]. Transgene expression was extinguished in condensing mesenchyme and chondrocytes and confined to periosteum and tendons at E15.5. After birth the mesenchymal cells in periosteum showed *Prx1*-driven expression [50]. The *Prx1*-expressing periosteal cells could differentiate into chondrocytes and osteoblasts *in vitro*. Therefore, the periosteal *Prx1*-expressing cells were most likely chondro-osteoprogenitor cells [51]. Thus, the *Prx1* promoter might be useful to study osteophyte formation that stems from the periost, or to study early phases of chondrogenesis.

#### ***Choosing the right promoter provides no guarantees***

Even though the name of a gene promoter suggests a similar expression pattern as the corresponding protein the gene is referring to, this is at best only an approximation. Not all regulatory elements upstream or downstream of the transcription start site have been fully specified. Manipulating the lengths of promoter and intronic regions can further specify or generalize expression patterns. It is worthwhile further characterizing each element to generate the perfect promoter. Therefore, it is very likely that transgenic promoters will become more elegant in the future. Based on chondrocyte-specific promoters that have been extensively studied, it has become evident that binding of SOX9 is a key element in driving chondrocyte specificity.

*Sox9* itself, however, is not a chondrocyte-specific promoter. Even though the *Sox9*-cre reporter also drove expression in limb bud mesenchymal cells giving rise to chondrocytes and osteoblasts, it also drove expression in spinal cord, epithelium of the intestine, pancreas and mesenchyme of the testis [52].

One has to keep in mind that the expression levels achieved in transgenic mouse models are not solely due to the promoter/enhancer regions that drive the transgene as different sites of integration can severely influence the level of expression. This can be due, for example, to methylation or simply the activity of the area in the genome that influences accessibility of the transgene itself. Comparing all of the different promoters studied by different groups clearly shows that the choice of a combination of regulatory elements as well as the number of copies used greatly influence the level and specificity of expression. For instance, the aggrecan promoter used by Doege and colleagues [22] showed weak expression, whereas that used by Han and Lefebvre [23] showed strong expression *in vivo*. This could be due to the longer DNA fragment Doege and colleagues used that could negatively influence expression, but could also be due to the four copies of the A1 fragment Han and Lefebvre used. Unfortunately, careful choice of elements provides no guarantees. Fujimaki and colleagues [17] showed that different founder mice using the same construct of *Col11a1*-cre displayed different expression patterns, which could be due to physical or functional shortening of the promoter region during transgene integration or position effects at the site of integration. Some founders even showed no expression at all despite integration of the transgene.

### Inducibility

Use of the promoters described above is a great solution to circumvent embryonic lethality and other major problems of general overexpression or deletion. However, studying the expression of a factor at a certain stage of development or even in adulthood requires an additional level of regulation: adding an 'on' or 'off' switch to enable regulation in time.

#### ***Cre-loxP***

Cre-loxP recombination was discovered in the early 1980s in bacteria and is a very powerful tool for generating transgenic mice. Sauer [53] reviewed the cre-loxP system for transgenic mice in 1998. Cre recombinase, originating from the P1 bacteriophage, is the crucial enzyme in this system. It belongs to the integrase family of site-specific recombinases [54] and catalyzes recombination between two loxP recognition sites. loxP consists of an 8 bp core sequence that is flanked by two 13 bp inverted repeats, where the asymmetric core defines the orientation of the loxP site. One recombinase molecule binds to each palindromic half of a loxP site and then forms a tetramer to bring the two loxP sites together, where the core loxP site undergoes recombination. Depending on the orientation of the loxP site, the recombination will result in an excision, inversion, insertion or translocation of the DNA [54]. It can thereby function as a loss-of-function, but also as a gain-of-function tool, either by insertion or excision of a stop codon.

##### 

**Use of cre-loxP** Combining the cre-loxP system with tissue-specific promoters provides a highly flexible system with the unique opportunity to disrupt or overexpress genes in a tissue-specific manner. However, this is still limited by the expression patterns of the chosen promoters/enhancers. As soon as a promoter is activated, so is the transgene. In general this is fine when the goal is to investigate the effect in chondrocytes generally, but when the goal is to investigate a factor at a specific stage, such as adulthood, or even during ageing, additional problems appear: how to restrict expression to a certain period of time. In 2006 Grover and Roughley [55] took the modulation of chondrocyte specificity to the next level and added the component necessary to do this. They generated a transgenic mouse that allowed cartilage-specific cre recombinase expression under control of the *Col2a1* promoter, but added tetracycline-controlled transcriptional activation to add a level of control over the timing of expression [55]. Since then, similar systems have been developed for many other promoters as well.

#### ***Flp-FRT***

The Flp-FRT system is quite similar to the cre-loxP system. Flp (flippase), which is named for its ability to 'flip' a DNA segment, recombines the short flippase recognition target (FRT) sites [56]. Like the LoxP site, FRT consists of two 13 bp palindromic sequences and an 8 bp asymmetric core and basically functions the same way.

The main difference between both systems is that cre-LoxP originated in bacteria, whereas Flp-FRT is of yeast origin. The preference for cre-LoxP is due to its bacterial origin as it functions optimally at 37°C whereas Flp-FRT has an optimal temperature of 30°C. However, both are optional and have been used to generate transgenic animals for both knock-out and gain-of function purposes.

##### 

**LOFT** New applications of the cre-LoxP and Flp-FRT systems combine LoxP with Frt called a LoxP-FRT trap (LOFT) to enable generation of reversible knock-outs [57]. These use two alleles of a target gene: a floxed allele and an allele with a FRT-flanked gene-trap cassette that inactivates the target gene while expressing green fluorescent protein (GFP). The latter is inactive, but convertible into a wild-type allele. Using cre, it is thus possible to first delete the target gene and thereafter restore its expression again by using Flp. This system is of course not limited to inactivation or deletion, but can be used for activation of a transgene as well.

#### ***Tet system***

Tetracycline-controlled transcriptional activation is a way to induce gene expression where transcription is turned on (Tet-on) or off (Tet-off) in the presence of tetracycline or one of its derivatives, of which doxycycline (dox) is usually the preferred choice. Regulation in this Tet-system is both reversible and quantitative due to use of varying concentrations of dox. The system consists of a transactivator and a transactivator-dependent promoter. The Tet-off system is composed of a tetracycline-controlled transactivator protein (tTA) and a tetracycline-responsive promoter element (TRE). The transactivator is composed of a Tet repressor DNA binding protein (tetR) derived from the tetracycline resistance operon *tet* of *Escherichia coli* transposon Tn10 fused to the tet repressor and activating domain of VP16 of the herpes simplex virus [58]. The TRE is composed of a tet operator sequence (tetO) fused to a minimal promoter. In the absence of dox, tTA will bind TRE and activate transcription. In the Tet-on system, a reverse tetracycline-controlled activator (rtTA) is present instead of the tTA. This rtTA has a four amino acid change in tetR, resulting in recognition of tetO only in the presence of dox [59].

##### 

**Use of the tet system** The combination of a Tet-off or Tet-on system with tetracycline can provide the flexibility to stop or activate, respectively, transgene expression at any given point in time for any desired period. Grover and Roughley [55] made a construct containing the *Col2a1* promoter driving rtTA that undergoes a conformational change, allowing it to bind to the tetO element that was present within the same construct, coupled to a minimal cytomegalovirus (CMV) promoter. When transgenic embryos were exposed to dox, this resulted in cre expression in isolated spines and ribs. Also, pups from mothers exposed to dox showed cartilage-specific expression, but no expression in liver, brain or skin. Also in 1-month-old mice exposure to dox resulted in transgene expression in articular cartilage and growth plate. In 3-month-old mice transgene expression in the growth plate could no longer be detected, but it still could be in articular cartilage.

This opened up a wide array of new possibilities. Mice with mutations that were previously embryonically lethal could now be born alive and have the floxed mutation induced at any given point in time. Thus, typical problems like rib cages that are unstable due to interference with skeletal build up could be circumvented.

#### ***Cre-ER***^***T***^

Another way to add an on-switch to tissue-specific transgenic mice is by using tamoxifen-induced recombination. In this system, the cre gene is fused with a mutant ligand binding domain (LBD) of the human estrogen receptor (ER): cre-ER^T^[60]. This chimeric recombinase can be activated by the natural ligand of the LBD, in this case tamoxifen. Due to the mutation of the LBD, only the synthetic ligand can bind, thereby circumventing binding of natural ligands that activate the construct.

##### 

**Use of Cre-ER**^**T**^ Nakamura and colleagues [61] combined a cre-ER^T^ under the control of a *Col2a1* promoter. They reported that there was a thin line between level of expression and tamoxifen control as robustly expressing lines displayed rapid and complete recombination, but might have leakiness, whereas those that had tight regulation showed low expression. Chen and colleagues [62] produced an almost identical mouse and demonstrated nice expression in articular cartilage in 2-month-old mice, thereby clearly showing it is possible to initiate expression upon adulthood. The now widely used Cre/ER^T2^ mutant, containing the G400V/M543A/L544A triple mutant in the human ER LBD, was found 4- to 10-fold higher in induction compared with Cre/ERT [63]. Henry and colleagues [24] successfully used this CreER^T2^ combined with the above-mentioned aggrecan promoter to induce expression in adult cartilage.

##### 

**Tet system versus cre-ER**^**T**^ The benefit of the cre-ER^T^ system is that, in contrast to the tet system, it requires only a single exposure to enable recombination: once it is switched on, it will remain switched on. However, this can also be a limitation as removal of dox also implies removal of the transgene in the tet system. Thus, both systems have possible benefits over the other and the choice of system truly relies on its purpose.

#### ***Limitations of cre recombinase-driven tissue specificity***

Most studies characterizing expression in response to regulatory elements are performed in embryonic tissue, showing specific expression patterns during development. When using adult animals, however, the expression patterns can be different to these. Recently, Fosang and colleagues [64] published a study using the *Col2a1*-cre mouse crossed with a LacZ reporter mouse and showed that expression in adults was not restricted to chondrocytes. They proposed that this was most likely due to the origin of the synovial cells; their parental cells originated from the interzone and therefore had already experienced *Col2a1*-driven cre expression. The limitation of LacZ was removed due to excision of the floxed area. They suggest that, having already been exposed to cre recombinase, all daughter cells show the same phenotype, thereby losing cartilage tissue specificity during adulthood. Other groups have made similar proposals in unpublished data concerning osteoblasts, but whether or not this is the long-debated transdifferentation from cartilage to bone is still undecided by the scientific community. However, these findings impose a major limitation on a system that is now widely used as cartilage-specific. Therefore, it is crucial to include proper controls and regularly check tissue specificity in one’s own strains.

When using a tissue-specific promoter driving cre one has to keep in mind that the cre is always expressed. This system can get leaky over time or off-target effects can be found, for example, by pseudo-loxP sites that can be present in endogenous genes. Therefore, it is crucial to check tissue specificity in each strain regularly by crossing it with a reporter mouse to ensure that one’s specific strain is not subject to non-specific effects like those found by Fosang and colleagues.

A point to consider when using inducible systems is that a wide variety of options are available to activate them. Tetracycline, dox, tamoxifen and so on can be administered in many ways (in drinking water, food, by injections, and so on) and a wide variety of dosages can be used. These all influence uptake and therefore activity. In addition, they could have an effect on the model being studied. For instance, dox has been investigated in osteoarthritis (OA) and it has been suggested by some to have protective effects, although discussion is ongoing. Low dosages in mice, however, have not been found to affect cartilage damage, so it can still be used in OA models to study cartilage.

### Transgenesis techniques

Jaenisch and Mintz [65] made the first transgenic animal using random integration by microinjection of DNA into fertilized oocytes in 1974. In 2007 Capecchi, Evans and Smithies received the Nobel Prize 'for their discoveries of principles for introducing specific gene modifications in mice by the use of embryonic stem cells'; they used homologous recombination in embryonic stem cells [66]. The use of BAC clones has added the capacity to integrate large DNA constructs without the use of restriction sites [67].

Novel techniques are being continuously developed. For example, one can use lentiviruses to transfect embryos, resulting in random integration in the genome [68]. However, if the site of integration is an issue, zinc finger nucleases (ZFNs) or recombinase-mediated cassette exchange are possible options. ZFNs are artificial restriction enzymes that enable editing of the genome through a ZFN pair that targets a specific DNA site leading to a targeted gene deletion or transgene integration [69]. Recombinase-mediated cassette exchange replaces an existing gene cassette with an analogous one containing the transgene of interest [70].

A technique that could be of interest with regard to tissue specificity is knock-in of transgenes into 3' UTRs of endogenous genes to mimic their expression patterns. Several 3' UTR IRES-Cre mouse strains have been developed, most of which show similar expression patterns to the endogenous gene without affecting mRNA stability [71]. This method could potentially be used to introduce transgenes into 3’ UTRs of cartilage-specific genes.

### Inducible tissue-specific transgene expression in mature murine articular cartilage

Even though tissue-specific inducible transgenic mice are now common in research, they are not widely used to study the effect of transgenes in mature articular cartilage. Most researchers use them to study cartilage development rather than cartilage/meniscal maintenance and degeneration. A search on PubMed for 'cartilage specific inducible transgenic' or 'chondrocyte specific inducible transgenic' (to search for the cartilage-specific variant of tissue specificity) yielded 14 hits at the time of writing this review. Only three studies actually constructed an inducible cartilage-specific transgenic mouse. Others were non-inducible, not cartilage-specific or simply a non-applicable hit. The three hits included a study by Chen and colleagues [62] describing how they made the *Col2a1*-CreERT2 mouse, which was used in the other two studies. Of these two, only one actually performed experiments during adulthood [72]; the other investigated development [73]. This search is of course a limited view since not all potentially relevant manuscripts fit within these search criteria, but it does show that there is much still to be done in this field.

Weng and colleagues [72] inhibited fibroblast growth factor receptor 1 in adult articular cartilage by combining a floxed *Fgfr1* mouse with the *Col2a1-*CreERT2mouse and using intraperitoneal injection of tamoxifen at 8 weeks; success was confirmed by PCR. These mice were subjected to experimentally induced OA and rheumatoid arthritis or aged naturally and the authors were able to show that Fgfr1 was chondroprotective and associated with decreased MMP13 expression compared to wild-type mice [72].

We recently used a combination of the *Col2a1* promoter and the Tet-on system to generate a mouse (*Col2a1*-rtTA-BMP2) that successfully expressed BMP2 in articular chondrocytes during adulthood after induction of OA [74]. In this mouse, once a cell has expressed *Col2a1*, driven by the *Col2a1* promoter, cre recombinase is also expressed, resulting in recombination of the floxed rtTA, thus leading to rtTA expression. Only when the mice were exposed to dox did rtTA bind to the TRE-minCMV promoter that induces expression of BMP2. In contrast to the suggested limitations of *Col2a1*-induced expression with age, we did not encounter problems with expression in adulthood and have recently successfully tested expression in old mice as well (unpublished data). It is likely that due to prior induced *Col2a1*-driven recombination, constant *Col2a1* activity is not required to activate the Tet-on system in our transgenic mouse.

Strikingly, not all successfully generated mice have been reported in the literature. Dr A Lassar has generated a *Prg4*/GFP/Cre/ERt2 mouse that is currently in use and has been presented at conferences, but has not yet led to publications. PRG4 is a proteoglycan that is also known as lubricin and is expressed predominantly in the top layer of articular cartilage. This mouse (*Prg4*^*tm1(GFP/cre/ERT2)Abl*^/J; available at Jax Mice) expresses a GFP/cre/ERT2 fusion protein in the top layer of articular cartilage in younger mice and in both top and deep layers of articular cartilage in older mice.

The combination of cartilage specificity and inducibility offers great potential for studying complex diseases such as OA and rheumatoid arthritis. Especially for OA, where we still do not know exactly how and why cartilage and menisci degenerate and what we can do about it, these mice hold great promise.

## Conclusion

As described in this review, there are abundant choices and paths to take when making chondrocyte-specific (inducible) transgenic mice. Each promoter has a different expression pattern, but clearly SOX9 is pivotal in steering chondrocyte specificity. It seems that the *Col2a1* promoter is a great choice for high constant expression in all chondrocytes, whereas the *Col11a2* and *Matn1* promoters govern a more distinct subset of chondrocytes but with weaker expression. *Col10a1* can be used when expression specifically in hypertrophic chondrocytes is required. When chondrocyte specificity is of less importance, *Gdf5* or specific regions, the *Col6a1* promoter to drive expression in the entire joint or the *Prx1* promoter can be used to study limb development.

Altering the length of promoter regions as well as enhancer regions in introns has a major impact on expression patterns *in vivo*. Careful choice of regulatory elements provides no guarantee as the site of integration as well as the number of integrated copies greatly influence the spatiotemporal nature and level of expression. Therefore, developing and testing multiple transgenic founders will be crucial to gain a transgenic mouse line with optimal expression patterns. Fortunately, we currently have several well-characterized transgenic mice in which chondrocyte-specific promoters are coupled to cre recombinase. These flexible systems are regularly used with consistent outcomes. Combining them with an inducible component provides flexible systems for chondrocyte-specific expression at any given point in time with great potential for studying OA pathogenesis, subject to their limitations.

## Abbreviations

BAC: Bacterial artificial chromosome; bp: Base pair; CMV: Cytomegalovirus; dpc: Days post-coitum; dox: Doxycycline; E: Embryonic day; EMSA: Electrophoretic mobility shift assay; ER: Estrogen receptor; FRT: Flp recombination target; GFP: Green fluorescent protein; HMG: High mobility group; IRES: Internal ribosome entry site; LBD: Ligand binding domain; MATN: Matrilin; OA: Osteoarthritis; PCR: Polymerase chain reaction; RSC: Rat chondrosarcoma; rtTA: Reverse tetracycline-controlled activator; tetO: Tet operator sequence; tetR: Tet repressor DNA binding protein; TRE: Tetracycline responsive promoter element; tTA: Tetracycline-controlled transactivator protein; UTR: Untranslated region; ZFN: Zinc finger nuclease.

## Competing interests

The authors declare that they have no competing interests.
